# A clinical strategy to improve the diagnostic performance of 3T non-contrast coronary MRA and noninvasively evaluate coronary distensibility: combination of diastole and systole imaging

**DOI:** 10.1186/s12968-023-00982-5

**Published:** 2023-11-23

**Authors:** Hongfei Lu, Shihai Zhao, Di Tian, Yinyin Chen, Jianying Ma, Meiying Ge, Mengsu Zeng, Hang Jin

**Affiliations:** 1 Department of Radiology, Zhongshan Hospital, Fudan University and Shanghai Institute of Medical Imaging, No. 180 Fenglin Road, Shanghai, 200032 China; 2 Department of Radiology, Zhongshan Hospital (Minhang Meilong Branch), Fudan University and Shanghai Geriatric Medical Center, Shanghai, 200237 China; 3 Department of Cardiology, Zhongshan Hospital, Fudan University, Shanghai, 200032 China

**Keywords:** Coronary artery disease, Magnetic resonance angiography, 3T non-contrast, Diagnostic performance, Distensibility

## Abstract

**Background:**

The clinical application of coronary MR angiography (MRA) combining diastole and systole imaging has never been described comprehensively in coronary artery disease (CAD) patients. We aimed to design an optimal non-contrast coronary MRA scan protocol combining diastolic and systolic imaging and to (1) evaluate its diagnostic performance for detecting significant coronary stenosis; (2) evaluate the feasibility of this protocol to noninvasively measure the coronary distensibility index (CDI).

**Methods:**

From June 2021 to May 2022, 33 healthy volunteers and 91 suspected CAD patients scheduled for X-ray coronary angiography (CAG) were prospectively enrolled. 3T non-contrast water-fat coronary MRA was carried out twice at diastole and systole. Significant coronary stenosis was defined as a luminal diameter reduction of ≥ 50% using CAG as the reference and was evaluated as follows: (1) by coronary MRA in diastole alone; (2) by coronary MRA in systole alone; (3) by combined coronary MRA in diastole and systole. According to CAG, the patients were divided into significant CAD patients and non-significant CAD patients. The difference in CDI among participants was evaluated.

**Results:**

Combined coronary MRA was completed in 31 volunteers and 76 patients. The per-patient sensitivity, specificity, and accuracy of combined coronary MRA were 97.5%, 83.3%, and 90.8%, respectively. Compared with single diastolic mode, combined coronary MRA showed equally high sensitivity but improved specificity on a per-patient basis (83.3% vs. 63.9%, adjusted *P* = 0.013). The CDI tested by coronary MRA decreased incrementally from healthy volunteers to non-significant and significant CAD patients.

**Conclusion:**

Compared with single-phase mode, 3 T non-contrast combined coronary MRA significantly improved specificity and may have potential to be a simple noninvasive method to measure CDI.

**Supplementary Information:**

The online version contains supplementary material available at 10.1186/s12968-023-00982-5.

## Background

Cardiovascular diseases, including coronary artery disease (CAD), are the major cause of death worldwide [1]. For noninvasive assessment, coronary computed tomography angiography (CTA) provides high accuracy for detection of coronary artery stenoses in routine clinical practice [2]. Coronary magnetic resonance angiography (MRA) is a complementary noninvasive and radiation-free alternative for coronary anatomical evaluation with no need for contrast medium, but it has not been widely used in clinical practice [3] due to lower diagnostic accuracy than CTA [4, 5]. There are two relatively quiescent periods in the cardiac cycle, mid-diastole and end-systole, for acquiring coronary images [6]. Both coronary MRA [7–9] and CTA [10] protocols are usually set to acquire data or reconstruct images during the diastole of the cardiac cycle. In a study from 2005, Wu et al. [11] have found that 1.5T coronary MRA in systole offered superior image quality in patients with high heart rates (HR). Gharib et al. [12] have also demonstrated the potential of imaging the coronary arteries at 3T in systole. Thus, the combination of diastolic and systolic imaging may potentially enhance the clinical applications of 3T coronary MRA.

In addition to morphological changes, the biomechanical properties of coronary arteries such as vascular stiffness, have been reported as indicators of cardiovascular aging to predict future clinical events [13–16]. Coronary vessel distensibility, which is a reflection of arterial stiffness, is reduced with atherosclerosis and normal aging. Changes in lumen diameter or area during a cardiac cycle are usually used to test vascular distensibility, but most tests available for measuring the coronary distensibility are either invasive or associated with radiation exposure [17]. Recently, MR imaging has been introduced as a promising tool for assessing both morphological and biomechanical changes on coronary arteries in various patient groups [18, 19]. The two rest periods during the cardiac cycle allow us to acquire two sets of coronary artery images using disparate acquisition windows. Subsequently, the coronary distensibility index (CDI) can be calculated. Hence, in addition to detecting significant coronary stenoses, coronary MRA combining diastolic and systolic imaging may be a promising noninvasive method to measure coronary distensibility.

The purpose of this study was to design an optimal non-contrast coronary MRA imaging protocol combining diastole and systole imaging and to (1) evaluate its diagnostic performance for detecting significant CAD using X-ray coronary angiography (CAG) as a reference standard; (2) evaluate the feasibility of this protocol to noninvasively measure the CDI.

## Methods

### Participants

Our institutional Ethics Committee on clinical research approved the study and written informed consent was obtained from each participant. In this prospective study, from June 2021 to May 2022, 124 participants including 33 healthy volunteers and 91 patients with suspected CAD were enrolled consecutively for coronary MRA. Healthy volunteer inclusion criteria were as follows: (1) older than 18 years; (2) no history of cardiovascular disease; (3) concentrations of blood glucose, lipids, and creatinine all within the reference range; (4) no history of smoking; and (5) body mass index (BMI) within the reference range. Inclusion criteria for patients were as follows: (1) older than 18 years; (2) an intermediate to high risk of CAD and scheduled for CAG. We excluded participants with general contraindications to MRI (e.g., pacemakers, claustrophobia) and/or the following factors: atrial fibrillation, unstable angina, prior myocardial infarction, history of coronary stent, or coronary artery bypass surgery.

### Reference standard

CAG served as the reference standard in the current study, which was carried out in accordance with the guidelines of the American College of Cardiology [20]. The CAG images were evaluated by two cardiologists (J.Y.M, with 15 years of experience and reader 2, with 12 years of experience) in consensus. Significant coronary artery stenosis was defined as a luminal diameter reduction of ≥ 50% [21]. Stenoses were evaluated using quantitative coronary angiography (QCA; Siemens Healthcare, Erlangen, Germany) for segments with a reference diameter ≥ 1.5 mm. The American Heart Association (AHA) segmentation model was used [22]. A coronary vessel was defined as having relevant CAD if a stenosis ≥ 50% existed in any segment.

### Acquisition of whole-heart coronary MRA data

To avoid the effect on vascular function assessment, no contrast agents, additional beta-blockers or nitroglycerin (NTG) were used in the current study.

Whole-heart coronary MRA was carried out on a 3T scanner (Ingenia CX; Philips Healthcare, Best, the Netherlands). Firstly, a fast localization sequence was performed to identify the position of the heart and diaphragm. Four-chamber cardiac cine images of the heart were then obtained to assess the minimal motion phase of the right coronary artery (RCA). The non-contrast whole-heart free-breathing coronary MRA was performed by a 3D segmented Turbo Field gradient echo sequence. The Dixon water-fat separation technique was applied in this sequence. The compressed sensitivity encoding (CS-SENSE) technique with an acceleration factor of five was employed to speed up the data acquisition. The detailed parameters of coronary MRA are provided in Additional file 1.

Whole-heart coronary MRA was run twice with the same parameters (but different acquisition phases): once acquired during diastole and once acquired during systole. The two scans were performed in random order. For each participant, the peripheral blood pressure was obtained within 2 h before or after MR imaging, and the HR was monitored throughout scanning.

### Whole-heart coronary MRA image analysis

MR data were transferred to a workstation (IntelliSpacePortal Version 9.0.4; Philips Healthcare, Best, the Netherlands) for image reconstruction. The images were assessed independently by two radiologists (H.F.L., with 5 years of experience, and S.H.Z., with 7 years of experience) who were blinded to the CAG results. The coronary arteries were assessed according to the AHA segmentation model [22]. The image quality of each coronary artery segment was rated on the following four-point scale: 1 = poor, non-assessable with severe image artifacts; 2 = fair, assessable with moderate image artifacts; 3 = good, assessable with minor artifacts; and 4 = excellent, assessable with no apparent artifacts [21]. Segments that could not be visualized received an image quality score of 1. Scores of the two observers were averaged for image quality analysis.

In the current study, to avoid false negative results, the intention to read analysis model was used and non-assessable segments at coronary MRA were considered to have a stenosis [23–26]. Stenosis with diameter reduction ≥ 50% was defined as significant stenosis. Each observer independently determined the presence or absence of significant stenosis narrowing using the following three strategies: (1) by coronary MRA in diastole alone; (2) by coronary MRA in systole alone; (3) by combined coronary MRA (combining diastolic and systolic images). For combined coronary MRA analysis, only location-matched stenosis presented in both modes was determined to be significant, otherwise, it was defined as having no significant stenosis. Consensus reading was performed for the segments with disagreement between the two observers.

For evaluating coronary distensibility, cross-sectional images of coronary arteries were reconstructed by using multiplanar reconstruction on the basis of axial original-source MR images acquired at both diastole and systole. The coronary lumen area was calculated with the help of IntelliSpacePortal software and related details are provided in Additional file 1. The CDI was calculated on the basis of differences between coronary lumen areas with different acquisition phases. CDI (in millimeters of mercury^−1^) was defined as [(A_sys_ − A_dia_)/(A_dia_ × pulse pressure)] × 1000 [17], where A_sys_ and A_dia_ are the lumen area at systole and diastole, respectively. The first observer (H.F.L.) measured CDIs twice at two separate times to test the repeatability of CDI measurements at MRA.

### Statistical methods

Although results of two readers’ independent evaluations are presented separately, formal statistical comparison testing was performed for only the consensus or mean evaluations between the two readers. All statistical analyses were performed with SPSS version 26.0 (SPSS, Inc., Chicago, Illinois, United States). The normality of continuous variables was tested using the Shapiro–Wilk test. Quantitative variables were expressed as mean value ± standard deviation (SD) if data were normally distributed, and nonnormally distributed data as median (interquartile range [IQR]). Categorical variables were presented as the frequency and percentage.

According to the outcomes of CAG, the patients were divided into significant CAD patients (with significant stenosis) and non-significant CAD patients (without significant stenosis). The characteristics between the significant and non-significant CAD patients were compared with X^2^ and unpaired Student *t* tests. Diastolic and systolic coronary MRA acquisition time, navigator efficiency, trigger to data acquisition time, and acquisition window of all study participants were compared using paired Student *t* tests. Image quality of coronary MRA at diastole and systole was presented as mean value ± SD [21], which were compared using the nonparametric paired Wilcoxon signed rank test. Pulse pressure among significant CAD patients, non-significant CAD patients, and healthy volunteers were compared with one-way ANOVA analysis. If there were differences, Tukey or Games–Howell post-hoc tests were applied.

The diagnostic performance of coronary MRA for the detection of significant coronary artery stenosis (sensitivity, specificity, positive predictive value (PPV), negative predictive value (NPV), and accuracy) was calculated on a per-patient, per-vessel, and per-segment basis. The Cochran Q test was used to compare the sensitivity, specificity and accuracy among diastolic, systolic and combined coronary MRA on a per-segment, per-vessel and per-patient basis. For three paired groups with significant results, between-group differences were compared by using post-hoc Dunn tests with Bonferroni corrections. Coronary lumen area and CDI results of the three groups of participants were compared with one-way ANOVA analysis, followed by Tukey or Games–Howell test.

Interobserver agreement for stenosis analysis was evaluated on a per-patient basis by using unweighted kappa. For CDI, interobserver agreement was evaluated by the intraclass correlation coefficients (ICC). A two-tailed *P* value < 0.05 was considered significant.

## Results

### Characteristics and key MR parameters of the study participants

**Fig. 1:**
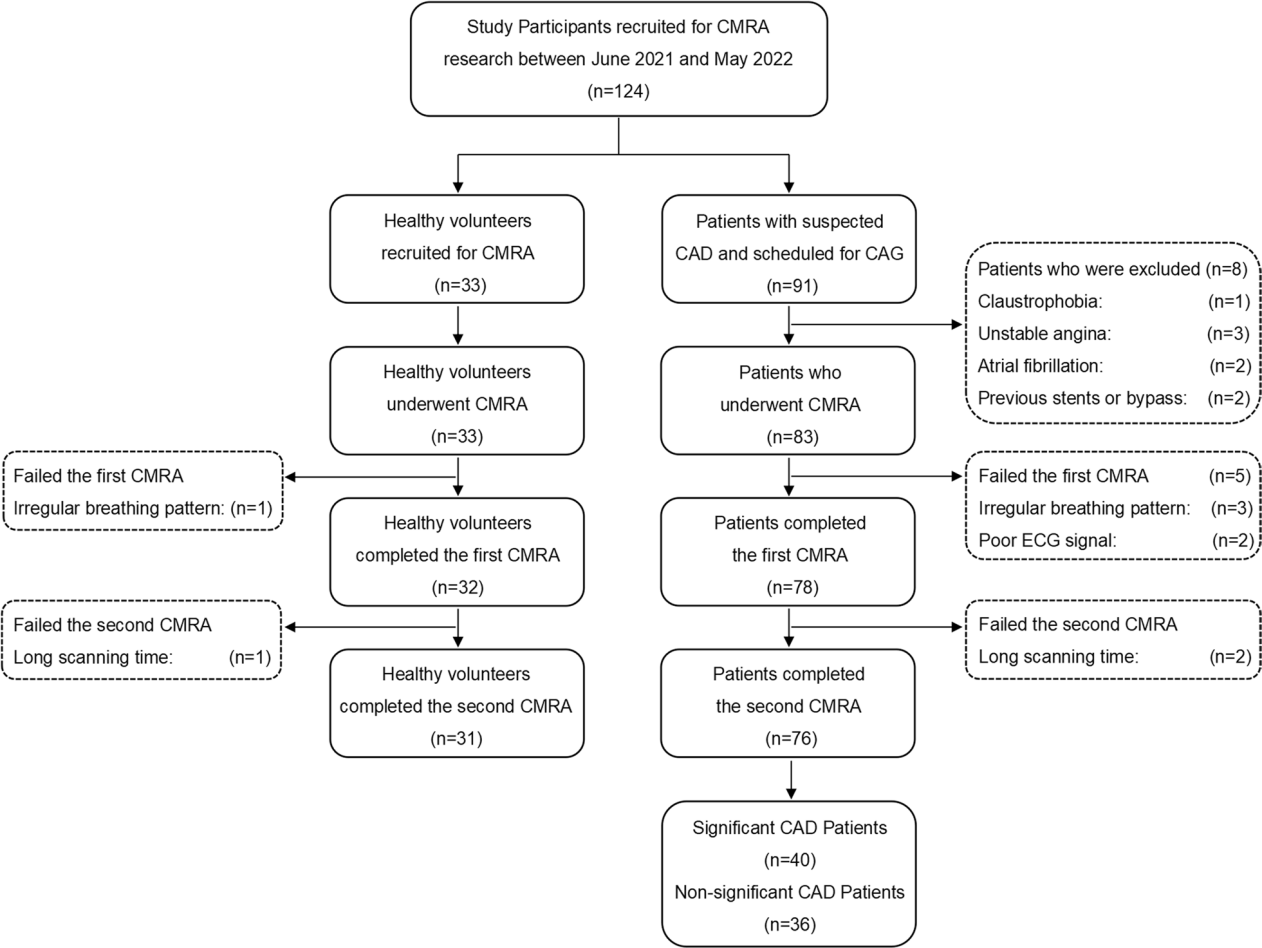
Flowchart of the study participants. *CMRA* coronary magnetic resonance angiography, *CAD* coronary artery disease, *CAG* X-ray coronary angiography, *ECG* electrocardiography

The study participant inclusion flowchart is shown in Fig. 1. Diastolic and systolic coronary MRA was successfully completed in 31 volunteers and 76 patients. All 76 patients underwent CAG within one week after coronary MRA. The detailed reasons for exclusion and failed coronary MRA are listed in Fig. 1. The pulse pressures of significant CAD patients, non-significant CAD patients, and healthy volunteers were significantly different (*P* < 0.001), and the pulse pressure in healthy volunteers was lower than that in significant CAD patients (*P* < 0.001) and non-significant CAD patients (*P* = 0.001). Study participants’ characteristics are summarized in Table 1.

**Table 1:** Characteristics of the 107 Study Participants

Characteristic	Significant CAD patients (n = 40)	Non-significant CAD patients (n = 36)	Healthy volunteers (n = 31)	*P* value
Sex (male:female)	31: 9	20: 16	16: 15	0.042
Age (y)^a^	61 ± 7	58 ± 9	26 ± 2	0.093
Age range (y)	39—74	36—73	24—30	NA
Heart rate^a^	67 ± 8	70 ± 13	70 ± 8	0.243
Body mass index (kg/m^2^)^a^	26.0 ± 4.6	23.9 ± 3.0	21.6 ± 1.9	0.025
LVEF (%)^a^	64 ± 6	66 ± 7	66 ± 3	0.425
Systolic blood pressure (mm Hg)^a^	131 ± 16	123 ± 15	110 ± 8	0.029
Diastolic blood pressure (mm Hg)^a^	79 ± 10	75 ± 10	71 ± 7	0.131
Pulse pressure (mm Hg)^a^	52 ± 13	48 ± 12	39 ± 7	0.130
No. with hypertension	29	14	0	0.003
No. with hypercholesterolemia	18	14	0	0.590
No. with diabetes mellitus	7	4	0	0.429
No. of current or prior cigarette smokers	15	7	0	0.083

Data are number of participants except where otherwise indicated

*CAD* coronary artery disease, *LVEF* left ventricular ejection fraction

*P* value: significant CAD patients vs. non-significant CAD patients

^*^Data are mean ± standard deviation

No significant difference was found between diastolic and systolic coronary MRA in navigator efficiency (57 ± 10% vs. 58 ± 10%, *P* = 0.079). The trigger to data acquisition time (576 ± 84 ms vs. 262 ± 45 ms, *P* < 0.001), and acquisition window (144 ± 46 ms vs. 99 ± 16 ms, *P* = 0.009) were significantly different between diastolic and systolic coronary MRA. The total scan time for the entire coronary MRA imaging protocol was 16.9 ± 3.5 min. The coronary MRA acquisition time was longer at systole compared with that at diastole (9.4 ± 2.0 min vs. 7.5 ± 2.2 min, *P* < 0.001).

### Image quality of coronary MRA at diastole and systole

In the 31 volunteers, 368 segments could be visualized in diastole on coronary MRA and 370 segments in systole. In the 76 patients, 949 segments had a reference luminal diameter ≥ 1.5 mm on CAG images. In diastole, 934 of 949 segments were assessable (image quality score = 2–4) on coronary MRA and 938 of 949 segments were assessable in systole. No water-fat swap artifacts were observed in all coronary MRA images. In 107 study participants, the comparison of image quality between diastolic and systolic coronary MRA are shown in Table 2. The overall image quality of coronary MRA in systole was higher than that in diastole (*P* < 0.001), particularly in middle-distal segments: the middle, PDA/PL of RCA; the middle, distal, DA1, DA2 of LAD (*P* < 0.05 for all), and the image quality of other segments at systole was similar with that at diastole. The image quality results of two readers’ independent evaluations are provided in Additional file 1: Table S1.

**Table 2:** Comparison of image quality between coronary MRA at diastole and systole in 107 study participants

Parameter	No. of all segments	At diastole		At systole		*P* value
		Image quality	No. of segments with a score ≥ 3	Image quality	No. of segments with a score ≥ 3	
Overall	1319	3.63 ± 0.63	1238	3.72 ± 0.56	1265	< 0.001
RCA						
Proximal	107	3.83 ± 0.42	105	3.85 ± 0.38	106	0.640
Middle	105	3.44 ± 0.60	99	3.54 ± 0.64	101	0.063
Distal	104	3.58 ± 0.63	96	3.63 ± 0.58	99	0.426
PDA/PL	93	3.59 ± 0.66	81	3.74 ± 0.53	89	0.013
LM	107	3.95 ± 0.21	107	3.96 ± 0.19	107	0.566
LAD						
Proximal	107	3.92 ± 0.31	106	3.95 ± 0.21	107	0.207
Middle	107	3.78 ± 0.46	105	3.89 ± 0.32	107	0.007
Distal	107	3.34 ± 0.75	95	3.68 ± 0.58	103	< 0.001
DA1	96	3.49 ± 0.83	85	3.65 ± 0.67	88	0.018
DA2	74	3.51 ± 0.80	66	3.65 ± 0.65	69	0.017
LCX						
Proximal	107	3.87 ± 0.39	105	3.87 ± 0.34	107	> 0.99
Distal	101	3.36 ± 0.66	93	3.43 ± 0.75	89	0.276
OM	104	3.42 ± 0.76	95	3.49 ± 0.79	93	0.145

No. of all segments: including all segments could be visualized in 31 volunteers and segments with lumen diameter ≥ 1.5 mm on CAG images in 76 patients

Data are mean ± standard deviation

*MRA* magnetic resonance angiography, *LM* left main coronary artery, *RCA* right coronary artery, *LAD* left anterior descending coronary artery, *LCX* left circumflex coronary artery, *PDA* posterior descending artery, *PL* posterolateral branch, *DA1* the first diagonal branch, *DA2* the second diagonal branch, *OM* oblique marginal branch

### Diagnostic performance of coronary MRA using the three strategies

**Fig. 2:**
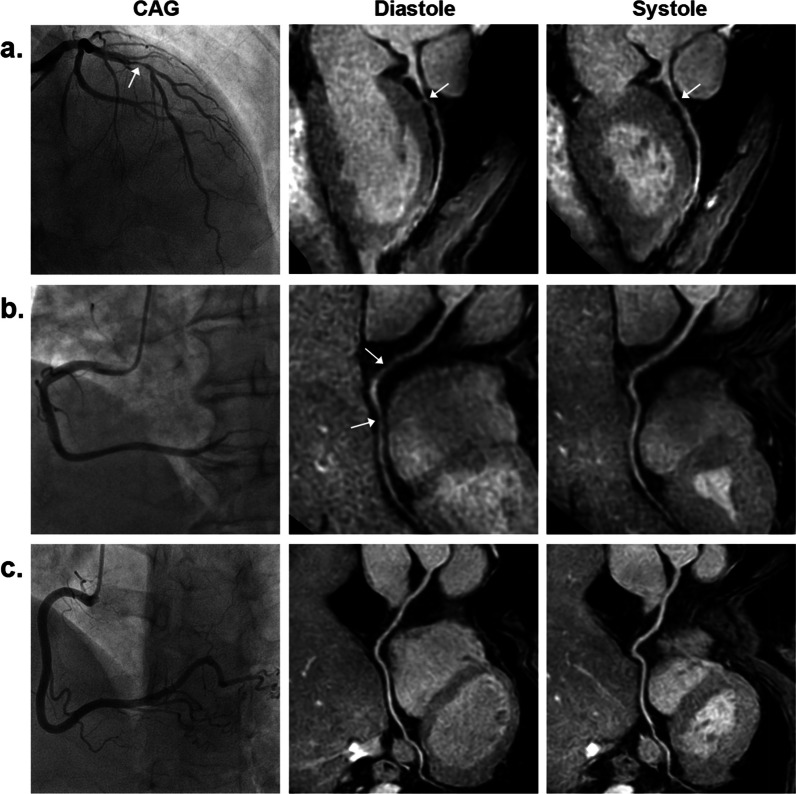
X-ray coronary angiography (CAG) and coronary MR angiography (MRA) at diastole and systole. **a** Left anterior descending artery (LAD) of a 68-year-old man: CAG shows a significant stenosis (arrow) in the proximal LAD. Coronary MRA at diastole and systole also show a significant stenosis (arrow) in the proximal LAD. For combined coronary MRA analysis, these two location-matched stenosis presented in both modes was determined to be significant. **b** Right coronary artery (RCA) of a 65-year-old woman: CAG shows a normal RCA without significant stenosis. Coronary MRA at diastole shows false-positive stenoses (arrows) of RCA, which were not observed at coronary MRA at systole, therefore the RCA was defined as having no significant stenosis. **c** LAD of a 54-year-old woman: CAG shows a normal LAD without significant stenosis. Both coronary MRA at diastole and systole show a normal LAD without significant stenosis, therefore the LAD was defined as having no significant stenosis

The sensitivity, specificity, PPV, NPV, and accuracy of coronary MRA for detecting significant CAD using the three coronary MRA strategies are listed in Table 3. Thirteen non-significant CAD patients were regarded as false positives at diastolic CMRA. Nine non-significant CAD patients were regarded as false positives at systolic CMRA. Seven of these false positive patients were corrected at combined CMRA. For the three coronary MRA strategies (diastolic, systolic and combined), there was no significant difference in sensitivity on a per-patient (*P* > 0.99), per-vessel (*P* = 0.174), and per-segment (*P* = 0.093) basis, while the specificity and accuracy were significantly different on a per-patient (*P* < 0.05 for both), per-vessel (*P* = 0.001, < 0.001 respectively), and per-segment (*P* < 0.001 for both) basis. Compared with diastolic coronary MRA, systolic coronary MRA had similar specificity and accuracy on a per-patient (adjusted *P* = 0.307 for both), per-vessel (adjusted *P* > 0.99 for both), and per-segment (adjusted *P* = 0.137, 0.088 respectively) basis. Compared with diastolic coronary MRA, combined coronary MRA had significantly higher specificity and accuracy on a per-patient (adjusted *P* < 0.05 for both), per-vessel (adjusted *P* < 0.05, = 0.001 respectively), and per-segment (adjusted *P* < 0.001 for both) basis. No significant difference was found between combined and systolic coronary MRA in specificity and accuracy on a per-patient basis (adjusted *P* = 0.662 for both), while these two indexes of diagnostic performance of combined coronary MRA were significantly higher than that of systolic coronary MRA on a per-vessel (adjusted *P* < 0.05 for both), and per-segment (adjusted *P* = 0.001 for both) basis. Figure 2 illustrates the diagnostic performance of coronary MRA combining diastole and systole with CAG as reference standard. The diagnostic results of two readers’ independent evaluations are provided in Additional file 1: Table S2). The kappa value for interobserver agreement for coronary artery stenosis detection with diastolic, systolic and combined coronary MRA was 0.883, 0.890, 0.867 respectively.

**Table 3:** Diagnostic performance of 3T non-contrast whole-heart coronary MRA at diastole and systole in 76 patients

Method	Sensitivity	Specificity	PPV	NPV	Accuracy
Per patient					
At diastole	97.5 (39/40)	63.9 (23/36)	75.0 (39/52)	95.8 (23/24)	81.6 (62/76)
At systole	97.5 (39/40)	75.0 (27/36)	81.3 (39/48)	96.4 (27/28)	86.8 (66/76)
Combined	97.5 (39/40)	83.3 (30/36)	86.7 (39/45)	96.8 (30/31)	90.8 (69/76)
Intergroups* P* value*	> 0.99	0.016	NA	NA	0.016
Adjusted* P* value^a^	NA	0.307	NA	NA	0.307
Adjusted* P* value^b^	NA	0.013	NA	NA	0.013
Adjusted* P* value^c^	NA	0.662	NA	NA	0.662
Per vessel					
At diastole	92.5 (62/67)	83.9 (135/161)	70.5 (62/88)	96.4 (135/140)	86.4 (197/228)
At systole	91.0 (61/67)	84.5 (136/161)	70.9 (61/86)	95.8 (136/142)	86.4 (197/228)
Combined	88.1 (59/67)	92.6 (149/161)	83.1 (59/71)	94.9 (149/157)	91.2 (208/228)
Intergroups* P* value*	0.174	0.001	NA	NA	< 0.001
Adjusted* P* value^a^	NA	> 0.99	NA	NA	> 0.99
Adjusted* P* value^b^	NA	0.003	NA	NA	0.001
Adjusted* P* value^c^	NA	0.007	NA	NA	0.003
Per segment					
At diastole	86.3 (88/102)	93.4 (796/852)	61.1 (88/144)	98.3 (796/810)	92.7 (884/954)
At systole	84.3 (86/102)	94.8 (808/852)	66.2 (86/130)	98.1 (808/824)	93.7 (894/954)
Combined	81.4 (83/102)	97.3 (829/852)	78.3 (83/106)	97.8 (829/848)	95.6 (912/954)
Intergroups* P* value*	0.093	< 0.001	NA	NA	< 0.001
Adjusted* P* value^a^	NA	0.137	NA	NA	0.088
Adjusted* P* value^b^	NA	< 0.001	NA	NA	< 0.001
Adjusted* P* value^c^	NA	0.001	NA	NA	0.001

Data are percentages (raw data)

For three paired groups (diastole, systole and combined) with significant results by using Cochran Q test, between-group differences were compared by using post-hoc Dunn tests with Bonferroni corrections

*MRA* magnetic resonance angiography, *PPV* positive predictive value, *NPV* negative predictive value

*P* Value*: statistical significance of three paired groups

Adjusted *P* value^a^: diastole vs. systole

Adjusted *P* value^b^: combined vs. diastole

Adjusted *P* value^c^: combined vs. systole

### Coronary distensibility measured by coronary MRA

**Fig. 3:**
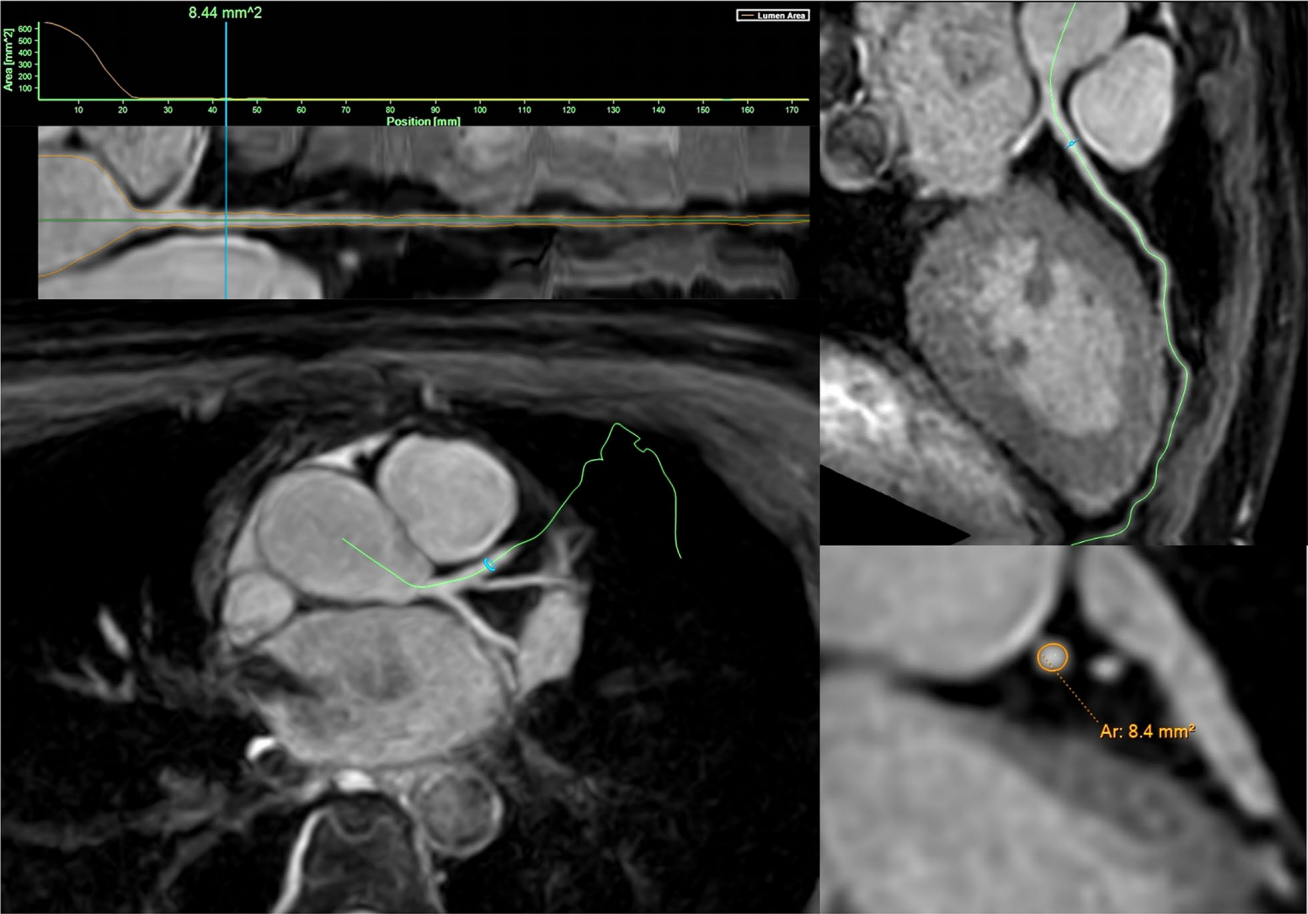
An example of IntelliSpacePortal cross-sectional coronary lumen measurement

An example of IntelliSpacePortal cross-sectional coronary lumen measurement is shown in Fig. 3. The Lumen area and CDI measured by coronary MRA are listed in Table 4. There was a significant difference in overall diastolic and systolic lumen area of the major coronary arteries among significant CAD patients, non-significant CAD patients and healthy volunteers (*P* < 0.001 for both). The overall diastolic lumen area of healthy volunteers was smaller than that of significant CAD patients and non-significant CAD patients (*P* < 0.001 for both), as well as the overall systolic lumen (*P* = 0.001, < 0.001 respectively). There was no significant difference in overall diastolic lumen area between significant CAD patients and non-significant CAD patients (*P* = 0.615), while the overall systolic lumen area of non-significant CAD patients was larger than that of significant CAD patients (*P* = 0.005). Similar results were observed for the RCA, LM, LAD and LCX lumen area.

**Table 4:** Comparison of coronary distensibility among the significant CAD patients, non-significant CAD patients and healthy volunteers

Parameter	Significant CAD patients (n = 40)	Non-significant CAD patients (n = 36)	Healthy volunteers (n = 31)	Intergroups *P* value*	*P* value^a^	*P* value^b^	*P* value^c^
LM							
Lumen area (mm^2^)							
At diastole	12.4 ± 4.1	13.3 ± 5.9	8.9 ± 3.1	< 0.001	0.715	< 0.001	0.001
At systole	13.4 ± 4.4	16.2 ± 7.4	11.3 ± 3.7	0.002	0.133	0.075	0.003
CDI (mm Hg^−1^)	1.7 ± 1.5	4.9 ± 2.8	8.1 ± 5.3	< 0.001	< 0.001	< 0.001	0.012
LAD							
Lumen area (mm^2^)							
At diastole	7.8 ± 2.7	8.7 ± 3.7	6.3 ± 2.4	0.005	0.477	0.039	0.007
At systole	8.6 ± 2.9	10.8 ± 4.2	8.6 ± 3.3	0.010	0.019	1.000	0.029
CDI (mm Hg^−1^)	2.0 ± 1.6	5.7 ± 3.0	10.1 ± 5.8	< 0.001	< 0.001	< 0.001	0.001
LCX							
Lumen area (mm^2^)							
At diastole	7.4 ± 2.6	7.7 ± 2.8	5.2 ± 1.9	< 0.001	0.881	< 0.001	< 0.001
At systole	8.3 ± 3.0	9.6 ± 3.5	6.9 ± 2.3	0.002	0.235	0.069	0.001
CDI (mm Hg^−1^)	2.4 ± 2.0	5.4 ± 3.4	9.5 ± 5.4	< 0.001	< 0.001	< 0.001	0.002
RCA							
Lumen area (mm^2^)							
At diastole	10.2 ± 4.4	10.0 ± 4.3	6.2 ± 1.8	< 0.001	0.982	< 0.001	< 0.001
At systole	11.0 ± 4.6	12.2 ± 5.1	7.9 ± 2.2	< 0.001	0.526	0.001	< 0.001
CDI (mm Hg^−1^)	1.6 ± 1.2	5.2 ± 3.2	8.1 ± 5.6	< 0.001	< 0.001	< 0.001	0.004
Overall							
Lumen area (mm^2^)							
At diastole	9.5 ± 4.0	9.9 ± 4.8	6.7 ± 2.7	< 0.001	0.615	< 0.001	< 0.001
At systole	10.3 ± 4.3	12.2 ± 5.8	8.7 ± 3.3	< 0.001	0.005	0.001	< 0.001
CDI (mm Hg^−1^)	1.9 ± 1.6	5.3 ± 3.1	8.9 ± 5.5	< 0.001	< 0.001	< 0.001	< 0.001

Data are mean ± standard deviation

*CAD* coronary artery disease, *CDI* coronary distensibility index, *LM* left main coronary artery, *LAD* left anterior descending coronary artery, *LCX* left circumflex coronary artery, *RCA* right coronary artery

Intergroups *P* value*: statistical significance of three groups

*P* value^a^: Significant CAD patients vs. non-significant CAD patients

*P* value^b^: Healthy volunteers vs. significant CAD patients

*P* value^c^: Healthy volunteers vs. non-significant CAD patients

**Fig. 4:**
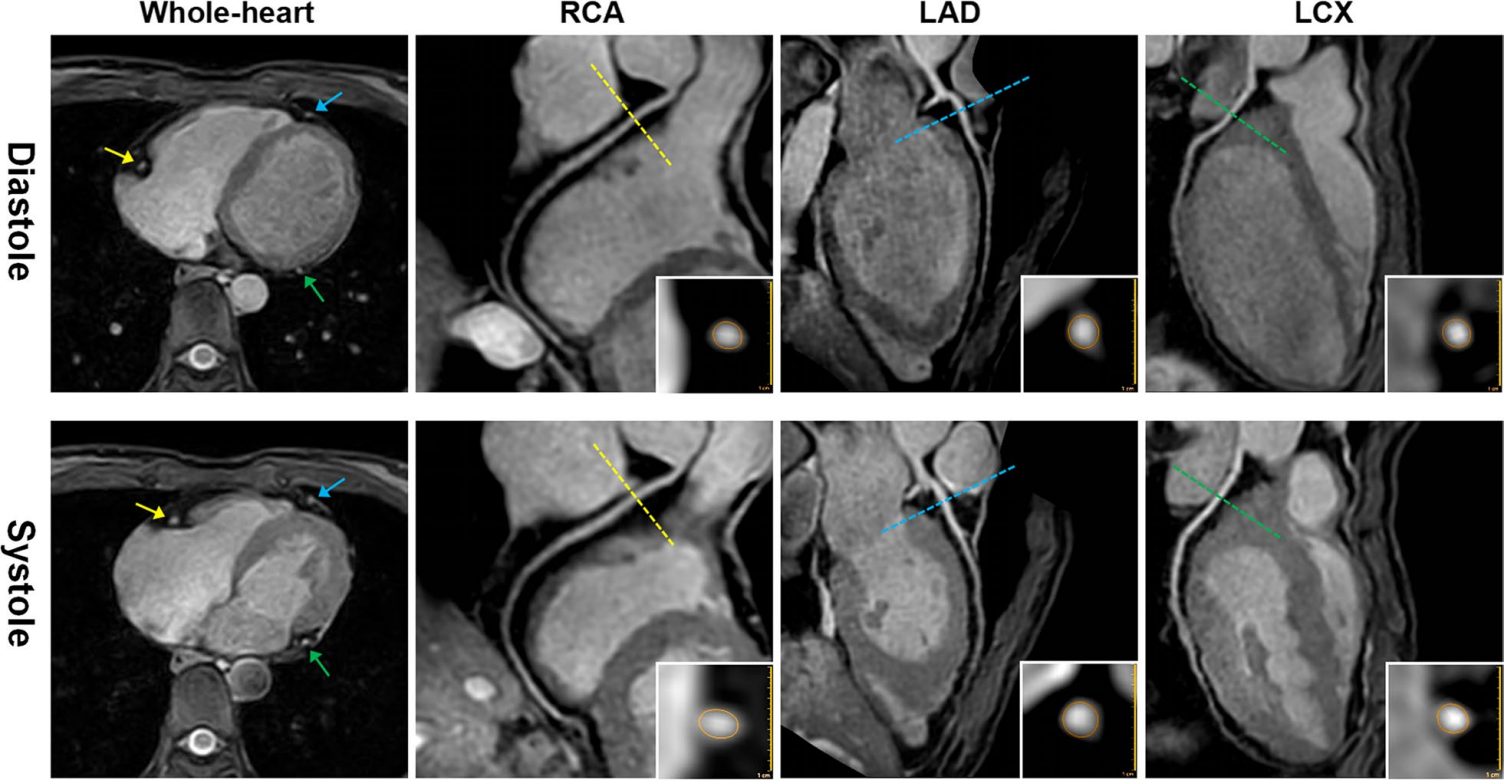
Coronary distensibility of a healthy volunteer measured with coronary MR angiography (MRA). Images in a 26-year-old female healthy volunteer. Peripheral blood pressure was 108/68 mm Hg (pulse pressure = 40 mm Hg). The axial original whole-heart MR images at diastole and systole show the difference in lumen size of right coronary artery (RCA) (yellow arrows), left anterior descending artery (LAD) (blue arrows),and left circumflex coronary artery (LCX) (green arrows). Coronary MRA images show remarkable coronary distensibility in RCA (lumen area 5.89 mm^2^ at diastole and 7.80 mm^2^ at systole, coronary distensibility index (CDI) = [(7.80 − 5.89)/(5.89/40)] × 1000 = 8.11 mm Hg^−1^), LAD (lumen area 7.01 mm^2^ at diastole and 9.73 mm^2^ at systole, CDI = [(9.73 − 7.01)/(7.01/40)] × 1000 = 9.70 mm Hg^−1^) and LCX (lumen area 5.02 mm^2^ at diastole and 6.93 mm^2^ at systole, CDI = [(6.93 − 5.02)/(5.02/40)] × 1000 = 9.51 mm Hg^−1^)

**Fig. 5:**
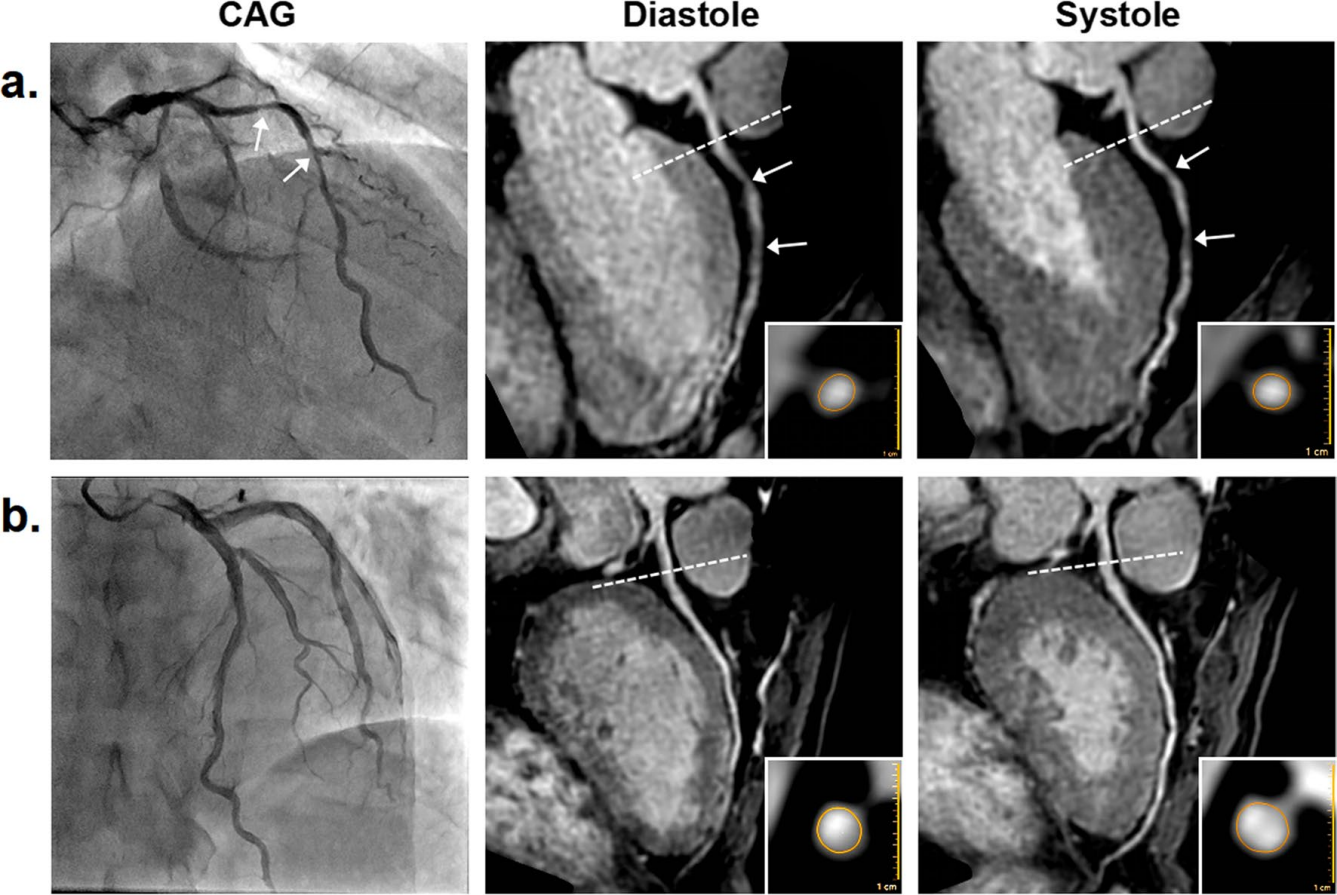
Coronary distensibility of two suspected coronary artery disease (CAD) patients measured with coronary MR angiography (MRA). **a** Images in a 55-year-old male suspected CAD patients. Peripheral blood pressure was 140/92 mm Hg (pulse pressure = 48 mm Hg). The X-ray coronary angiography (CAG) image shows significant stenoses in the proximal and middle left anterior descending artery (LAD). Coronary MRA images show slight coronary distensibility in LAD: lumen area 7.28 mm^2^ at diastole and 7.89 mm^2^ at systole, coronary distensibility index (CDI) = [(7.89 − 7.28)/(7.28/48)] × 1000 = 1.75 mm Hg^−1^. **b** Images in a 63-year-old male suspected CAD patients. Peripheral blood pressure was 134/90 mm Hg (pulse pressure = 44 mm Hg). CAG shows a normal LAD without significant stenosis. Coronary MRA images show moderate coronary distensibility in LAD: lumen area 11.79 mm^2^ at diastole and 14.60 mm^2^ at systole, CDI = [(14.60 − 11.79)/(11.79/44)] × 1000 = 5.42 mm Hg^−1^

For the three participants groups, there was a significant difference in overall CDI (*P* < 0.001). The overall CDI in both significant CAD patients and non-significant CAD patients were lower than that in healthy volunteers (*P* < 0.001 for both). Furthermore, compared with non-significant CAD patients, the overall CDI in significant CAD patients was also lower (*P* < 0.001). Similar results were observed for the RCA, LM, LAD and LCX CDIs. Typical examples of coronary distensibility detected by coronary MRA are shown in Figs. 4, 5. The CDI results of two readers’ independent evaluations are provided in Additional file 1: Table S3. CDI measurements showed excellent intraobserver agreement (ICC = 0.947) and good interobserver agreement (ICC = 0.884).

## Discussion

The major findings of this study are summarized as follows: (1) 3T non-contrast water-fat separated whole-heart coronary MRA in either diastole or systole can noninvasively detect CAD with high sensitivity and moderate specificity on a per-patient analysis; (2) combining diastolic and systolic imaging improved the diagnostic accuracy of water-fat separatd coronary MRA, especially specificity; and (3) CDI as measured with water-fat separated coronary MRA decreased incrementally from healthy volunteers to non-significant and significant CAD patients.

3T coronary MRA has usually been carried out with contrast agents [27, 28]. No intravenous contrast agents were used in the current study. Currently, more advanced fat suppression techniques have been proposed to improve coronary MRA image quality at 3T [29–31]. Our study applied Dixon water-fat separation technique to suppress epicardial fat signals around coronary arteries. We chose a wider receiver bandwidth for the Dixon sequence to reduce chemical shifts artifacts [32] and no water-fat swaps artifacts was observed in our study. Larger receiver bandwidth usually reduces SNR, but this is compensated for by the dual echo acquisition and the noise averaging effect taking place in the Dixon reconstruction [33]. A previous study has found that the Dixon water-fat method provides improved non-contrast coronary MRA image quality at 3T compared to the conventional spectral inversion recovery (SPIR) technique [32]. In a study from 2022, Lu et al. have demonstrated the feasibility of 3T non-contrast water-fat separated coronary MRA for detect clinically significant coronary stenosis [34]. There are two relatively quiescent periods in the cardiac cycle for acquiring images, mid-diastole and end-systole, lasting for an average of 187 ms and 118 ms [6] respectively. In the current study, we found that systolic imaging offered better image quality in middle-distal segments, however, the mean imaging time of coronary MRA was longer at systole than that at diastole due to differences in acquisition window. In the current study, we found higher sensitivity but lower specificity in diastolic or systolic mode alone, with no significant difference between diastole and systole. For coronary CTA, administration of NTG could help improve diagnostic performance and the visibility of the coronary arteries. However, the NTG may increase HR in some patients and affect image quality. Therefore, a beta blocker is often used simultaneously in patients with high HR to reduce the HR. To avoid the effect on vascular function assessment, no additional beta-blockers or NTG were used in our coronary MRA study. The HR of participants in our study were relatively stable, with less fluctuations. Compared with diastole, the duration of systole is less affected by HR variability [12]. End-systolic imaging may be an alternative to more conventional diastolic imaging to minimize the adverse effects of RR variability. However, the abbreviated systolic rest period necessitates image data collection in a relatively short acquisition window, which prolongs scanning time. The long acquisition time increases the chance of respiratory pattern drift, bulk motion, and all degrade the final image quality. Therefore, both methods have their own advantages and disadvantages, and both could be potentially helpful for clinical applications of coronary MRA. However, in our study, when diastole and systole images were combined, more false positive interpretations for poor image quality and ambiguous local artifacts were corrected, thereby enhancing the diagnostic specificity.

The coronary tree keeps deforming in a 3D space [17]. Therefore, most of the 2D MR techniques (e.g., 2D cine) [35] that have been widely used in other arteries (e.g. in the aorta, carotid and brachial arteries), cannot be directly applied to coronary arteries. Weissman et al. [36] measured the coronary lumen at end diastole and at early, mid-, and end-systole with intravascular ultrasonography. They found that the coronary luminal diameter increased 2.1% and luminal area increased 8.1% during mid- and late systole in 32 CAD patients [36]. Taking advantage of dual rest periods of cardiac motion, in a previous study, Lin et al. [17] successfully assessed CDI in older adults with 1.5T coronary MRA. They assessed 23 asymptomatic patients with diabetes mellitus with mean CDI of 2.79 ± 2.12 mm Hg^−1^ and 50 healthy aging subjects with mean CDI of 9.14 ± 5.87 mm Hg^−1^. Without signs or symptoms of cardiovascular disease, there was no CAG conducted as reference standard in their study. In our study, we recruited healthy volunteers and suspected CAD patients (divided into significant and non-significant CAD patients with CAG as the reference) for noninvasively coronary distensibility assessment without the application of medications. Our study reported comparable coronary distensibility to Lin’s study. In our study, the CDI was calculated on the basis of lumen area measurement, which was conventionally usually obtained with invasive Intravascular ultrasound imaging as the reference standard. Considering burden to participants, we did not conduct this invasive test in our subjects. Although the CAG was carried out in the suspected CAD patient, this examination is more suitable for measuring the lumen diameter and could not measure the lumen area directly. In addition, the contrast medium applied in CAG can cause coronary vasodilation [37] and the catheters used in intravascular ultrasound imaging can stimulate the endothelium [38], resulting in vasoconstriction. These factors may affect accurate assessment of CDI. Previous studies have proved that measurement of the coronary lumen area is more accurate with MRA than with conventional CAG [39, 40]. The quantitative image analysis software used in our study could also help accurate measurement of lumen area.

## Limitations

In our study, we proposed a clinical strategy of combining diastole and systole imaging to improve the diagnostic accuracy of 3T coronary MRA and noninvasively evaluate coronary distensibility for the first time. Our study still has several limitations. First, since this study reports a single-center experience, the sample size was relatively small. A further study with a larger sample size should be carried out. Second, there is non-matching between volunteers and patients, although appropriate statistical methods were applied to obtain relatively scientific results. Finally, combined coronary MRA mode need additional acquisition time due to combination of diastole and systole imaging, although the total time of coronary MRA was < 20 min. However, the non-contrast coronary MRA protocol is noninvasive, radiation and iodinated contrast-free, safe for repetitive acquisition.

## Conclusions

In conclusion, 3T non-contrast whole-heart water-fat separated coronary MRA using a combination of diastole and systole imaging was demonstrated to detect significant coronary artery stenosis with a sensitivity of 97.5% and specificity of 83.3% on a per-patient basis. Compared with single-phase coronary MRA mode, combined coronary MRA at 3T significantly improved diagnostic performance, especially specificity. Besides, this strategy may have potential to be a simple noninvasive method to measure coronary distensibility and evaluate coronary artery stiffness.

### Supplementary Information

**Additional file 1. **Supplementary methods and Tables.

## Data Availability

The data underlying this article will be shared on reasonable request to the corresponding author.

## Abbreviations:

CAD: Coronary artery disease
MRA: Magnetic resonance angiography
CTA: Computed tomography angiography
CAG: X-ray coronary angiography
CDI: Coronary distensibility index
