# Supplementary material for: A clinical strategy to improve the diagnostic performance of 3T non-contrast coronary MRA and noninvasively evaluate coronary distensibility: combination of diastole and systole imaging
Source: J Cardiovasc Magn Reson. 2023 Nov 23;25:67. doi: 10.1186/s12968-023-00982-5 (PMC10666457; doi:10.1186/s12968-023-00982-5)
Supplement: Supplementary file 1 — Additional file 1. Supplementary methods and Tables. [file 12968_2023_982_MOESM1_ESM.docx]

**Additional data**

**A clinical strategy to improve the diagnostic performance of 3.0-T non-contrast coronary MRA and noninvasively evaluate coronary distensibility: combination of diastole and systole imaging**

**Detailed parameters of coronary MRA**

**The coronary lumen area measurement details**

**Table S1**

**Table S2**

**Table S3**

**Detailed parameters of coronary MRA**

Whole-heart coronary MRA was carried out on a 3T scanner (Ingenia CX; Philips Healthcare, Best, the Netherlands) equipped with a 32-channel body phased-array surface coil and quantum gradients (maximum strength, 80 mT/m; slew rate, 200 (mT.m^-1^)/msec). The ECG triggering was performed using a four-lead electrocardiogram. Firstly, a fast localization sequence was performed to identify the position of the heart and diaphragm. Four-chamber cardiac cine images of the heart using a segmented 2D balanced steady state free precession (b-SSFP) sequence were then obtained to assess the minimal motion phase of the right coronary artery (RCA). The non-contrast whole-heart free-breathing coronary MRA was performed by a 3D segmented Turbo Field gradient echo sequence using the following parameters: TR/TE1/TE2, 4.4/1.42/2.6 ms; flip angle, 10°; FOV, 270 × 330 × 120 mm; acquisition matrix, 180 × 220 × 80; reconstructed matrix, 410 × 500 × 160; acquired spatial resolution, 1.50 × 1.50 × 1.50 mm; reconstructed spatial resolution, 0.66 × 0.66 × 0.75 mm. The Dixon water-fat separation technique was applied in this sequence. Bandwidth = 1232 Hz/pixel. A T_2_ preparation pulse (TE T_2_prep = 30 ms) was added to suppress the myocardial and venous blood signals. Real-time respiratory navigation with a gating window of ± 2.5 mm was achieved through a cardiac pencil-beam navigator perpendicular to the right diaphragm. The compressed sensitivity encoding (CS-SENSE) technique with an acceleration factor of five was employed to speed up the data acquisition.

**The coronary lumen area measurement details**

For the two sets of images, cross-sectional views at the major coronary arteries, including the left main (LM) artery and the proximal segments (10-15mm from the origin) of the RCA, the left anterior descending (LAD) artery, and the left circumflex (LCX) artery, were carefully matched for the surrounding anatomy and the distance from the coronary origin to the selected position. Once the cross-sectional plane was defined, coronary lumen contour was determined automatically using IntelliSpacePortal software and corrected manually by the two radiologists when necessary. Coronary lumen area was automatically calculated according to contours. If the proximal segment appeared to have stenosis, we would choose the normal proximal site away from the stenosis to measure coronary lumen area.

| **Table S1** | | | | | | | | | |
| --- | --- | --- | --- | --- | --- | --- | --- | --- | --- |
| **Image Quality of Coronary MRA at Diastole and Systole in 107 Study Participants Evaluated by the Two Readers** | | | | | | | | | |
| Image quality |  | At diastole | | |  | At systole | | |  |
|  |  | Average | Reader1 | Reader2 |  | Average | Reader1 | Reader2 |  |
| Overall |  | 3.63 ± 0.63 | 3.63 ± 0.63 | 3.63 ± 0.64 |  | 3.72 ± 0.56 | 3.72 ± 0.57 | 3.73 ± 0.55 |  |
| RCA |  |  |  |  |  |  |  |  |  |
| Proximal |  | 3.83 ± 0.42 | 3.81 ± 0.44 | 3.85 ± 0.38 |  | 3.85 ± 0.38 | 3.86 ± 0.37 | 3.84 ± 0.39 |  |
| Middle |  | 3.44 ± 0.60 | 3.47 ± 0.59 | 3.41 ± 0.65 |  | 3.54 ± 0.64 | 3.53 ± 0.65 | 3.55 ± 0.62 |  |
| Distal |  | 3.58 ± 0.63 | 3.58 ± 0.63 | 3.58 ± 0.63 |  | 3.63 ± 0.58 | 3.62 ± 0.58 | 3.63 ± 0.58 |  |
| PDA/PL |  | 3.59 ± 0.66 | 3.59 ± 0.65 | 3.59 ± 0.68 |  | 3.74 ± 0.53 | 3.72 ± 0.54 | 3.76 ± 0.52 |  |
| LM |  | 3.95 ± 0.21 | 3.95 ± 0.21 | 3.95 ± 0.21 |  | 3.96 ± 0.19 | 3.96 ± 0.19 | 3.96 ± 0.19 |  |
| LAD |  |  |  |  |  |  |  |  |  |
| Proximal |  | 3.92 ± 0.31 | 3.92 ± 0.31 | 3.92 ± 0.31 |  | 3.95 ± 0.21 | 3.95 ± 0.21 | 3.95 ± 0.21 |  |
| Middle |  | 3.78 ± 0.46 | 3.79 ± 0.46 | 3.77 ± 0.47 |  | 3.89 ± 0.32 | 3.87 ± 0.37 | 3.91 ± 0.29 |  |
| Distal |  | 3.34 ± 0.75 | 3.32 ± 0.77 | 3.36 ± 0.72 |  | 3.68 ± 0.58 | 3.66 ± 0.58 | 3.70 ± 0.57 |  |
| DA1 |  | 3.49 ± 0.83 | 3.49 ± 0.82 | 3.49 ± 0.85 |  | 3.65 ± 0.67 | 3.63 ± 0.68 | 3.67 ± 0.63 |  |
| DA2 |  | 3.51 ± 0.80 | 3.49 ± 0.80 | 3.54 ± 0.78 |  | 3.65 ± 0.65 | 3.66 ± 0.65 | 3.64 ± 0.65 |  |
| LCX |  |  |  |  |  |  |  |  |  |
| Proximal |  | 3.87 ± 0.39 | 3.85 ± 0.41 | 3.89 ± 0.37 |  | 3.87 ± 0.34 | 3.88 ± 0.33 | 3.86 ± 0.35 |  |
| Distal |  | 3.36 ± 0.66 | 3.38 ± 0.65 | 3.34 ± 0.67 |  | 3.43 ± 0.75 | 3.41 ± 0.75 | 3.45 ± 0.74 |  |
| OM |  | 3.42 ± 0.76 | 3.43 ± 0.76 | 3.41 ± 0.76 |  | 3.49 ± 0.79 | 3.50 ± 0.80 | 3.48 ± 0.78 |  |

**Note.** — Data are mean ± standard deviation. MRA = magnetic resonance angiography, LM = left main coronary artery, RCA = right coronary artery, LAD = left anterior descending coronary artery, LCX = left circumflex coronary artery, PDA = posterior descending artery, PL = posterolateral branch, DA1= the first diagonal branch, DA2= the second diagonal branch, OM= oblique marginal branch.

| **Table S2** | | | | | |
| --- | --- | --- | --- | --- | --- |
| **Diagnostic Performance of 3.0-T Non-Contrast Whole-Heart Coronary MRA at Diastole and Systole in 76 Patients Evaluated by the Two Readers** | | | | | |
| Method | Sensitivity | Specificity | PPV | NPV | Accuracy |
| Per Patient |  |  |  |  |  |
| At diastole (Consensus) | 97.5 (39/40) | 63.9 (23/36) | 75.0 (39/52) | 95.8 (23/24) | 81.6 (62/76) |
| Reader1 | 95.0 (38/40) | 61.1 (22/36) | 73.1 (38/52) | 91.7 (22/24) | 79.0 (60/76) |
| Reader2 | 97.5 (39/40) | 63.9 (23/36) | 75.0 (39/52) | 95.8 (23/24) | 81.6 (62/76) |
| At systole (Consensus) | 97.5 (39/40) | 75.0 (27/36) | 81.3 (39/48) | 96.4 (27/28) | 86.8 (66/76) |
| Reader1 | 97.5 (39/40) | 72.2 (26/36) | 79.6 (39/49) | 96.3 (26/27) | 85.5 (65/76) |
| Reader2 | 95.0 (38/40) | 75.0 (27/36) | 80.9 (38/47) | 93.1 (27/29) | 85.5 (65/76) |
| Combined (Consensus) | 97.5 (39/40) | 83.3 (30/36) | 86.7 (39/45) | 96.8 (30/31) | 90.8 (69/76) |
| Reader1 | 95.0 (38/40) | 80.6 (29/36) | 84.4 (38/45) | 93.6 (29/31) | 88.2 (67/76) |
| Reader2 | 95.0 (38/40) | 83.3 (30/36) | 86.4 (38/44) | 93.8 (30/32) | 89.5 (68/76) |
| Per Vessel |  |  |  |  |  |
| At diastole (Consensus) | 92.5 (62/67) | 83.9 (135/161) | 70.5 (62/88) | 96.4 (135/140) | 86.4 (197/228) |
| Reader1 | 89.6 (60/67) | 83.2 (134/161) | 69.0 (60/88) | 95.7 (134/140) | 85.1 (194/228) |
| Reader2 | 92.5 (62/67) | 83.9 (135/161) | 70.5 (62/88) | 96.4 (135/140) | 86.4 (197/228) |
| At systole (Consensus) | 91.0 (61/67) | 84.5 (136/161) | 70.9 (61/86) | 95.8 (136/142) | 86.4 (197/228) |
| Reader1 | 91.0 (61/67) | 83.9 (135/161) | 70.1 (61/87) | 95.7 (135/141) | 86.0 (196/228) |
| Reader2 | 89.6 (60/67) | 85.1 (137/161) | 71.4 (60/84) | 95.1 (137/144) | 86.4 (197/228) |
| Combined (Consensus) | 88.1 (59/67) | 92.6 (149/161) | 83.1 (59/71) | 94.9 (149/157) | 91.2 (208/228) |
| Reader1 | 86.6 (58/67) | 91.9 (148/161) | 81.7 (58/72) | 94.9 (148/156) | 90.4 (206/228) |
| Reader2 | 88.1 (59/67) | 92.6 (149/161) | 83.1 (59/71) | 94.9 (149/157) | 91.2 (208/228) |
| Per Segment |  |  |  |  |  |
| At diastole (Consensus) | 86.3 (88/102) | 93.4 (796/852) | 61.1 (88/144) | 98.3 (796/810) | 92.7 (884/954) |
| Reader1 | 85.3 (87/102) | 93.3 (795/852) | 60.8 (87/143) | 98.2 (795/811) | 92.5 (882/954) |
| Reader2 | 86.3 (88/102) | 93.4 (796/852) | 61.1 (88/144) | 98.3 (796/810) | 92.7 (884/954) |
| At systole (Consensus) | 84.3 (86/102) | 94.8 (808/852) | 66.2 (86/130) | 98.1 (808/824) | 93.7 (894/954) |
| Reader1 | 84.3 (86/102) | 94.7 (807/852) | 66.2 (86/130) | 98.1 (807/824) | 93.7 (893/954) |
| Reader2 | 83.3 (85/102) | 94.8 (808/852) | 65.9 (85/129) | 97.9 (808/825) | 93.6 (893/954) |
| Combined (Consensus) | 81.4 (83/102) | 97.3 (829/852) | 78.3 (83/106) | 97.8 (829/848) | 95.6 (912/954) |
| Reader1 | 80.4 (82/102) | 97.2 (828/852) | 77.4 (82/106) | 97.6 (828/848) | 95.4 (910/954) |
| Reader2 | 81.4 (83/102) | 97.2 (828/852) | 77.6 (83/107) | 97.8 (828/847) | 95.5 (911/954) |

Note. — Data are percentages (raw data). MRA = magnetic resonance angiography, CS-SENSE = compressed sensitivity encoding, PPV = positive predictive value, NPV = negative predictive value.

| **Table S3** | | | |
| --- | --- | --- | --- |
| **Coronary Distensibility Among the Significant CAD Patients, Non-significant CAD Patients and Healthy Volunteers Measured by the Two Readers** | | | |
| CDI (mm Hg^-1^) | Significant  CAD Patients  (n=40) | Non-significant  CAD Patients  (n=36) | Healthy  Volunteers  (n=31) |
| LM |  |  |  |
| Consensus | 1.76 ± 1.53 | 4.88 ± 2.83 | 8.06 ± 5.28 |
| Reader1-1st | 1.94 ± 1.73 | 4.77 ± 3.23 | 9.02 ± 5.63 |
| Reader1-2nd | 1.98 ± 1.86 | 4.56 ± 2.44 | 8.34 ± 5.29 |
| Reader2 | 1.58 ± 1.49 | 4.99 ± 2.91 | 7.10 ± 5.16 |
| LAD |  |  |  |
| Consensus | 1.99 ± 1.64 | 5.73 ± 2.98 | 10.09 ± 5.76 |
| Reader1-1st | 2.14 ± 1.62 | 6.34 ± 3.55 | 11.18 ± 6.65 |
| Reader1-2nd | 2.26 ± 1.55 | 6.15 ± 2.89 | 10.72 ± 6.12 |
| Reader2 | 1.84 ± 1.70 | 5.12 ± 2.58 | 9.01 ± 5.21 |
| LCX |  |  |  |
| Consensus | 2.38 ± 1.99 | 5.40 ± 3.38 | 9.53 ± 5.44 |
| Reader1-1st | 2.63 ± 2.17 | 5.20 ± 3.18 | 9.91 ± 6.03 |
| Reader1-2nd | 2.70 ± 2.16 | 5.27 ± 3.48 | 9.71 ± 5.13 |
| Reader2 | 2.14 ± 1.85 | 5.61 ± 3.92 | 9.14 ± 5.08 |
| RCA |  |  |  |
| Consensus | 1.63 ± 1.21 | 5.16 ± 3.15 | 8.05 ± 5.55 |
| Reader1-1st | 1.62 ± 1.25 | 5.62 ± 3.54 | 7.67 ± 4.87 |
| Reader1-2nd | 1.66 ± 1.14 | 5.32 ± 2.98 | 8.18 ± 4.62 |
| Reader2 | 1.63 ± 1.22 | 4.69 ± 2.89 | 8.66 ± 6.56 |
| Overall |  |  |  |
| Consensus | 1.94 ± 1.63 | 5.29 ± 3.07 | 8.93 ± 5.52 |
| Reader1-1st | 2.08 ± 1.75 | 5.48 ± 3.40 | 9.45 ± 5.90 |
| Reader1-2nd | 2.15 ± 1.75 | 5.32 ± 2.99 | 9.24 ± 5.36 |
| Reader2 | 1.80 ± 1.59 | 5.10 ± 3.10 | 8.48 ± 5.53 |

Note. — Data are mean ± standard deviation. CAD = coronary artery disease, CDI = coronary distensibility index. LM = left main coronary artery, LAD = left anterior descending coronary artery, LCX = left circumflex coronary artery, RCA = right coronary artery.
